# Gender and family-role portrayals of autism in British newspapers: An intersectional corpus-based study

**DOI:** 10.1177/13623613241303547

**Published:** 2024-12-11

**Authors:** Themis Karaminis, Costas Gabrielatos, Ursula Maden-Weinberger, Geoffrey Beattie

**Affiliations:** 1 Centre for Clinical, Social and Cognitive Neuroscience, Department of Psychology, City, University of London, UK; 2 Edge Hill University, UK

**Keywords:** autism, family, gender, newspapers, stereotypes, stigma

## Abstract

A recent large-scale study on the portrayal of autism in British newspapers revealed a deficit-based coverage, which concentrated on children and boys in particular, typically represented from the mothers’ perspective. This follow-up study refines these representations, considering how they differ by gender and family role. We analysed 2998 text samples, which discussed autism in the context of four combinations of gender and family roles, namely, BOY, GIRL, FATHER and MOTHER. These samples included sources with different publication dates, reporting style and political orientation. Autism representations remained negative regardless of gender and family role. Over time, stories about autistic girls started to emerge, identifying them as a distinct group explicitly compared to autistic boys. Newspapers, especially broadsheets, associated girls with diagnostic difficulties, camouflaging and sometimes gender dysphoria − discussed particularly for those assigned female at birth. The child’s autism was more often attributed to maternal than paternal behaviours or lifestyle. Autistic mothers were mentioned more often than fathers and were portrayed negatively. We conclude that newspapers portray female autism as less significant than male autism and, in addition, place mothers under more ethical scrutiny than fathers. These disparities reflect both historical biases in autism research and gender and family-role stereotypes.

**Lay abstract:**

News media influence how society views different social groups. A recent study which examined how British newspapers represent autism found that the coverage was largely negative, focused mainly on boys, and often presented their stories from the perspectives of their mothers. This follow-up study aims to understand how these representations vary by gender and family role. We analysed 2998 short newspaper texts discussing autism in terms of four groups: boys, girls, fathers and mothers. We looked at articles from different times, with various reporting styles and political leanings. Across all these sources, autism was portrayed negatively regardless of gender or family role. Over time, newspapers did tend to mention autistic girls more frequently, highlighting them as a distinct group compared to autistic boys. Newspapers, especially broadsheets, often focused on how autistic girls face difficulties in getting diagnosed and hide their characteristics, and on the role autism may play in gender identity issues, particularly for girls assigned female at birth. In addition, newspapers attributed more blame for the child’s autism to mothers’ than fathers’ behaviours. Autistic mothers were mentioned more frequently than fathers, however these mentions were often negative. Our results suggest that newspapers portray autism in girls as less significant than the autism of boys and criticise mothers of autistic children more harshly than their fathers. These findings reflect long-standing biases in autism research and reinforce broader stereotypes about gender and family roles. Such biased reporting may hinder public understanding and acceptance of the diverse experiences within the autistic community.

## Introduction

The diagnosis, research and understanding of autism have historically been deficit-based and heavily biased towards boys (Happé & Frith, 2020; Pellicano et al., 2022). Recently, research has begun to appreciate the diversity of the autistic experience and the importance of including and supporting all members of the autistic community. This shift is largely prompted by the neurodiversity movement, which underscores the need for intersectional perspectives, highlighting the overlap of autism with other social identities, to foster true acceptance and inclusion (Botha & Gillespie-Lynch, 2022; Grove et al., 2024; Mallipeddi & VanDaalen, 2022). However, stigma and a male-gender bias persist in public autism discourse, often coupled with broader stereotypes about disability, gender and a range of social categories (Draaisma, 2009; Holton et al., 2014; Karaminis et al., 2023). This study takes an intersectional approach to examine how representations of autism in the recent British press differ by gender and family roles. We would like to caution readers that this study includes selected quotes, some of which may be triggering.

### Gender and autism

Research consistently shows a higher prevalence of autism among males (Halladay et al., 2015; Volkmar et al., 1993), with the male-to-female ratio among autistic people estimated to be between 2:1 and 5:1 (Loomes et al., 2017; Lord et al., 2020). This gender disparity, also present in other types of neurodiversity, may reflect gender-specific differences in susceptibility to adverse prenatal and early developmental events (Nugent et al., 2018; Robinson et al., 2013). Regarding autism, the male-female prevalence disparity has been suggested to support two aetiological theories: the Empathising-Systematising theory (Baron-Cohen er al., 2005; Greenberg et al., 2018) and the extreme-male-brain theory (Baron-Cohen et al., 2011), which propose that autism represents an excessively masculine cognitive type characterised by a greater aptitude for analytical and rule-based systems (systemising) and a lower capacity to recognise others’ mental states (empathising).

However, there is a growing consensus that the relationship between biological sex, gender (a psycho-social construct; Goldman, 2013) and autism is more complex than previously thought (Happé & Frith, 2020; Lai et al., 2012; Mo et al., 2021; Strang et al., 2020). For example, a meta-analysis of autism prevalence studies found that studies applying autism screening procedures to general population samples tend to report a lower male-to-female ratio than studies using samples with a pre-existing autism diagnosis (Loomes et al., 2017). This discrepancy suggests that current diagnostic procedures may miss some autistic females (Loomes et al., 2017; see also D’Mello et al., 2022). Social camouflaging, the tendency to hide autistic characteristics in social situations, contributes to this underdiagnosis (Hull et al., 2017, 2021; Perry et al., 2022) and can lead to late diagnoses (Bargiela et al., 2016; Lundin et al., 2021; Whitlock et al., 2020) and adverse mental health outcomes (Leedham et al., 2020; Milner et al., 2019). Camouflaging has also been associated with the notion of the female autism phenotype (Bargiela et al., 2016; Hull et al., 2020), implying that autism manifests differently in males and females, beyond gender differences in the general population (Eriksson et al., 2012; Head et al., 2014).

Gender identity and gender dysphoria are also relevant to the understanding of the complex relationship between gender and autism. Autistic people, particularly those assigned female at birth, report weaker gender identification and self-esteem compared to neurotypical people and may pursue a gender transition (Cooper et al., 2022). Furthermore, transgender and gender-diverse people are more likely to exhibit higher levels of autistic characteristics or to have an autism diagnosis (Strauss et al., 2021; Warrier et al., 2020).

### Neurodiversity and intersectionality

Gendered autistic identities are gaining importance as the understanding of autism moves from medical and deficit-based views to embracing neurodiversity and social-based models (den Houting, 2019; Walker, 2012; Walker & Raymaker, 2020). This newer perspective recognises that autism can be a disability with biological and genetic underpinnings but emphasises the disabling effects of those environments that fail to support diverse ways of experiencing the world (Pellicano & den Houting, 2022). It also stresses the need to accept and represent all autistic people, acknowledging their unique capabilities that contribute to successful lives and broader societal progress (Ballou, 2018; Kapp et al., 2013; Pellicano et al., 2022). Crucially, full inclusion and representation require an intersectional approach, which accounts for how neurodiversity intersects with other identity aspects and social structures, such as gender, age, family-role, race and ethnicity (Botha & Gillespie-Lynch, 2022; Mallipeddi & VanDaalen, 2022). Intersectionality in this domain should not be limited to the neurodivergent people themselves but should include anyone with lived experience of neurodiversity, such as parents and families (Hall et al., 2022).

Under an intersectional lens, prominent theories and concepts in autism research can be challenged. For example, the notion of the female autism phenotype may highlight how research has overlooked the diversity of the autistic community across gender intersections. Similarly, the extreme-male-brain theory and the phenomenon of camouflaging may hinder the recognition and expression of gender diversity and non-conformity among autistic people, reinforcing existing power hierarchies. This results in female, trans and non-binary autistic people experiencing incongruence between their gender and their autistic identity (Coleman-Smith et al., 2020; Moore et al., 2022; Strang et al., 2018). However, people thrive only when their multiple identities are recognised and valued as a whole (Coleman-Smith et al., 2020; Koffer Miller et al., 2022).

### Gender and the representation of autism in the news media

The recognition and acceptance of gendered autistic identities can be hindered by stereotypes perpetuated in the public discourse (Draaisma, 2009), including fictional media (Jones et al., 2023; Mittmann et al., 2024; Nordahl-Hansen et al., 2018), news media and the press (Holton et al., 2014; Mittmann et al., 2024) and, more recently, social media (Corti et al., 2022; Gabarron et al., 2022; Skafle et al., 2021). News media and the press, in particular, mirror and help shape public attitudes towards social categories like autism and gender (Corrigan et al., 2005; Edelman, 1988; Hall Jamieson & Waldman, 2004; Happer & Philo, 2013). This influence is exercised through agenda setting, determining what is newsworthy, as well as framing and highlighting certain aspects of a topic.

With regard to the press, a growing body of studies indicates that newspapers often depict autism inaccurately, perpetuating ableist and stereotypical perspectives (Baroutsis et al., 2021; Billawala & Wolbring, 2014; Holton et al., 2014; Jones & Harwood, 2009). A common stereotype associates autism with children, especially boys (Akhtar et al., 2022; Huws & Jones, 2011; Karaminis et al., 2023). In addition, parents, especially mothers, are frequently the primary sources of information about autistic people, who lack agency in the described actions (Huws & Jones, 2011; Karaminis et al., 2023). This dual-gendered bias may reinforce ‘infantilising’ and ‘objectifying’ views (Botha & Cage, 2022; Stevenson et al., 2011) of autism, suggesting an interplay between negative attitudes towards autism and culturally mediated expectations for gender norms, roles, expressions and interests for boys and girls (Barnett, 1997; Davis & Hines, 2020; Witt, 1997), as well as for mothers and fathers (Hodges & Park, 2013).

The dual-gendered stereotype in the representation of autism was demonstrated quantitatively in a recent corpus-based study that analysed approximately 24,000 documents referring to autism in 10 major UK newspapers from 2011 to 2020 (Karaminis et al., 2023). One analysis in this study identified collocations of the terms, ‘autism’ and ‘autistic’. A collocation analysis identifies words (collocates) that co-occur with unusual frequently with the terms in focus (nodes, in our case, ‘autism’ and ‘autistic’) and lend some of their meaning to them (Sinclair, 1991). A prominent group of collocates included words like *son, child, boy, young, mother, girls, father, parent* and *families*, showing that newspapers focused on the gender and family relations of people who are autistic or associated with autism. The frequency of these collocations presented a strong bias towards children and boys. For example, the two most frequent gender-specific collocations, *son* and *boy*, were twice as frequent as *child*, the next most frequent collocation. In addition, *mother* was mentioned almost three times more often than *father*. This gender imbalance in collocations showed a slight decrease over time, and more recent newspapers also made references to transgender autistic people. Moreover, these changes were more pronounced in broadsheets than in tabloids, and in left-leaning rather than right-leaning newspapers.

To delve deeper into the autism representations that emerged from the quantitative procedures identifying collocations and to reveal explicit and implicit attitudes in context, Karaminis et al. (2023) conducted a qualitative discourse prosody analysis (Stubbs, 2001, p. 66). The authors manually annotated short text samples where the terms ‘autism’ and ‘autistic’ co-occurred with words associated with male children, such as *boy, son* and *lad*. By narrowing the focus to male autistic children, they were able to discern autism representations for the most prominent group of collocations related to gender and family roles. However, this focus meant that the discourse prosody analysis did not address how representations differ by gender and family role.

### Current study

This study explicitly examines gender-related disparities in the representation of autism in British newspapers. Using an intersectional approach, we conduct a systematic discourse prosody analysis of various text samples to enable two gendered comparisons. First, we contrast the representations of autistic boys and girls. Second, we consider gender differences beyond childhood, focusing on portrayals of mothers and fathers, who have lived experience of autism even if they themselves are not autistic. We hypothesise that this novel approach will offer new insights into how gender biases from autism research as well as broader stereotypes about gender and family roles shape press representations of autism.

## Method

### Materials

#### Text samples from the Autism UK Press Corpus

We analysed randomly selected text samples from the Autism UK Press Corpus (Karaminis et al., 2023). This corpus comprises 23,741 articles referencing autism or autistic people, published between 2011 and 2020 in 10 national UK newspapers: *Daily Express, Daily Mail, Daily Mirror, Daily Star, Daily Telegraph, Guardian, Independent, Observer, Sun* and *Times*. The corpus is organised as a complete collection (full corpus) and seven subcorpora based on reporting style (Tabloids: *Daily Express, Daily Mail, Daily Mirror, Daily Star, Sun*; Broadsheets: *Daily Telegraph, Guardian, Independent, Observer, Times*), political orientation (Left-leaning: *Daily Mirror, Guardian, Independent, Observer*; Right-leaning: *Daily Express, Daily Mail, Daily Star, Daily Telegraph, Sun, Times*) and publication period (Early: 2011−2014; Middle: 2015−2017; Recent: 2018−2020) (Karaminis et al., 2023; see also Balfour, 2019).

Text samples were chosen pseudo-randomly using WordSmith Tools 8 (Scott, 2021). Each sample included the terms ‘autism’ or ‘autistic’ and a word (collocate) related to gender and family roles, along with the 25 words before and after this word, totalling about 50 words per sample. For example, a sample including the collocate *girl* (in italics) was,

. . . loud noise. Yet she was diagnosed with autism only in February . . . ‘Girls are much better at fitting in than boys and their obsessions are very similar to non-autistic *girls*. For example, when I was a girl I was obsessed with soaps, like Neighbours and EastEnders. I knew all the characters, who played who, but apparently . . .’. [Guardian, May 2014]

#### Collocation groups

We defined four groups of collocations related to gender and family roles, namely, BOY, GIRL, FATHER and MOTHER. Each group included various semantically related words (see Table 1). For instance, the GIRL group included *girl, schoolgirl, daughter, granddaughter* and so on, along with their plural and possessive forms.

**Table 1.** The semantically related words which defined the four groups of collocations used to generate the intersecting sample sets. The joint frequency column corresponds to the total counts of the collocation, namely, how many times the terms ‘autism’ and ‘autistic’ co-occurred with the words in the list in the full corpus.

Collocation group	Total joint frequency	Collocations (joint frequency)
BOY	4210	boy (1305), schoolboy (33), lad (116), son (2727), grandson (29)
GIRL	1277	girl (684), schoolgirl (14), daughter (571), granddaughter (8)
FATHER	160	father (141), dad (15), grandfather (4), granddad (0)
MOTHER	650	mother (444), mum (206), grandmother (0)

#### Sets of text samples

We generated sets of 100 text samples for each of the four collocation groups, both within the full corpus as well as within the seven subcorpora. This process resulted in a total of 32 sample sets (four collocations groups from the full corpus and seven subcorpora), corresponding to an initial 3200 text samples, which was reduced to 2998 after removing duplicates and some samples that were deemed irrelevant, as they did not express any attitudes towards autism.

### Discourse prosody analysis

#### Annotation

Five members of the research team manually annotated sets of text samples. For each text sample, the annotators assigned values to five categories describing relationships, meanings, attitudes and discourses surrounding autism (see Figure 1):

**Figure 1.**
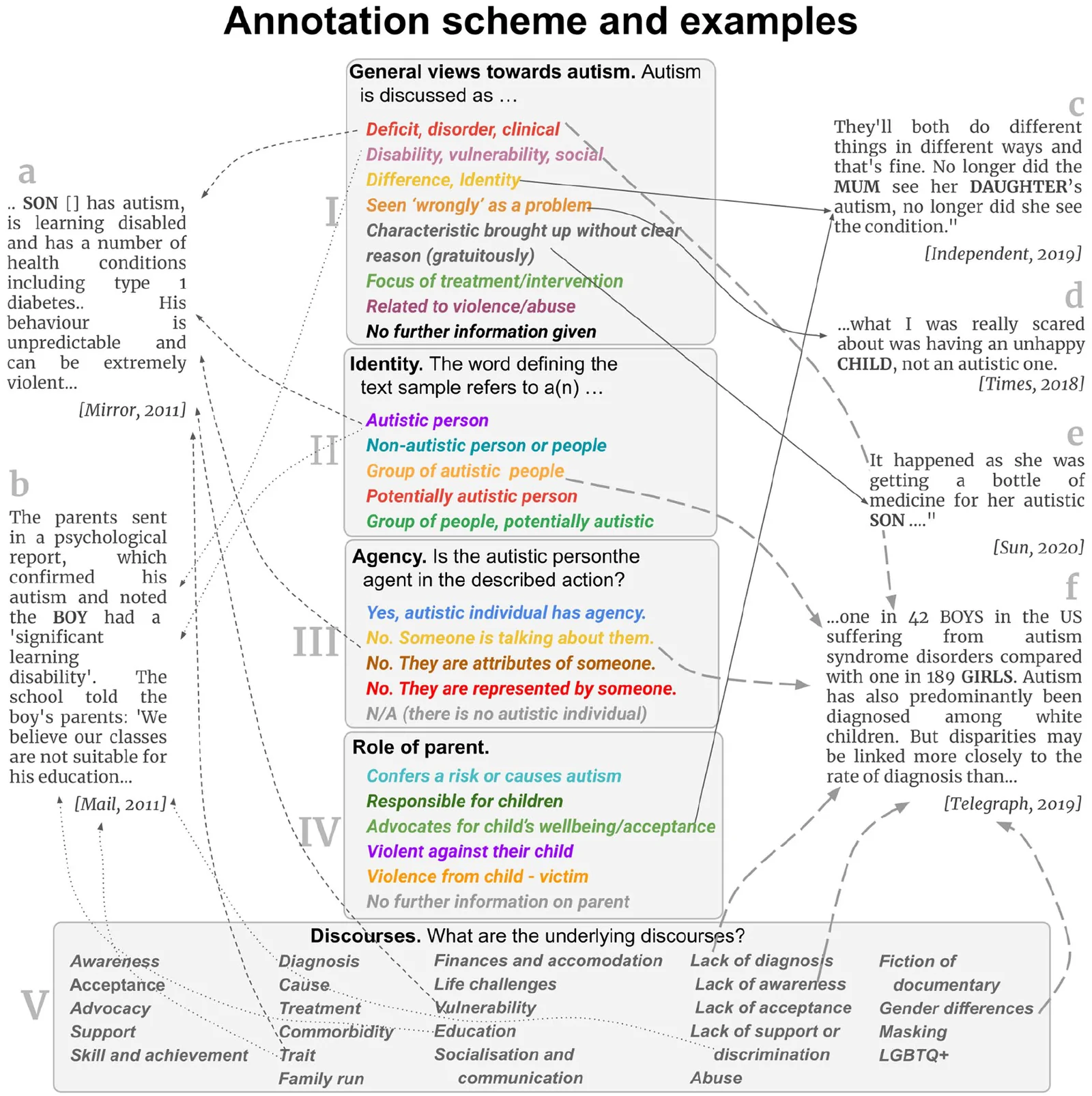
Annotation scheme and examples. The grey boxes show the five categories (I to V) and the values they take. Corpus samples *a, b* and *f* are examples of annotation. Only one value could be assigned to a sample from categories I–IV; for category V (discourses), multiple values could be assigned to the same sample. Corpus samples *c, d* and *e* are examples of the ‘Difference and not disability value’ and the ‘Mentioned gratuitously’ value of category I: General views towards autism.

1. I. General views towards autism: describing autism as a deficit, a disability or a difference.
2. II. Diagnostic status: identifying whether the BOY, GIRL, FATHER or MOTHER referred to in the sample was an autistic person, a non-autistic person, a group of autistic people, and so on.
3. III. Agency: determining whether an autistic BOY or GIRL had agency in the action described in the sample or was instead discussed or represented by someone.
4. IV. Role of parent: examining whether the MOTHER or FATHER was portrayed as someone conferring a risk for, or causing, someone’s autism, or as someone advocating or being responsible for their child, and so on.
5. V. Discourses: identifying the underlying discourses in the sample, for example, autism awareness, acceptance, education, and gender differences.

The annotation scheme expanded on Karaminis et al. (2023) by introducing category IV, relevant only to the FATHER and MOTHER sets. Single values were chosen for categories I to IV, while multiple values could be applied to category V. Annotators convened regularly to discuss their work and resolve differences, often referring to larger text sections or entire articles to clarify annotation issues.

#### Data analysis

After the annotation, we counted the frequency of each value within categories. We conducted pairwise quantitative comparisons between the proportion of a specific value in different sets of text samples. For example, we compared proportions for the values ‘autistic person’ and ‘group of autistic people’ between BOY and GIRL, or between MOTHER and FATHER sets. We used the Bayesian Information Criterion measure (BIC; Wilson, 2013, p. 8). A BIC ⩽ 0 indicates no difference; positive BIC < 2.00 indicates anecdotal evidence for a difference; BIC ⩾ 2.00 indicates positive evidence; BIC ⩾ 6.00 or ⩾10.00 indicates strong or very strong evidence, respectively.

For category V–Discourses, we measured the overall similarity of discourses between sets using correlation coefficients calculated over the 25 values in this category. These correlations were visually represented in heatmaps summarising discourse similarity between collocation groups, for example, BOY vs GIRL, in the full corpus and subcorpora. In addition, we generated heatmaps to show how discourses for a given set of text samples, for example, BOY, differed across newspapers with different reporting style, political orientation, or different publication times.

### Ethical approval

This research received ethical approval from the Science Research Ethics Committee of Edge Hill University (ScREC: ETH2021-0008) and was conducted in compliance with its ethical procedures.

### Community involvement statement

One autistic person (female postgraduate student), and two parents of autistic children (a father of an autistic boy, and a mother of an autistic girl) participated in the annotation of samples. One of these people further contributed to all stages of the research and is a co-author of this article.

## Results

### Category I: general views towards autism

Figure 2 presents the discourse prosody analysis results from the entire corpus. Panel A highlights the distribution of attitudes towards autism in the text samples. Across all four sets – BOY, GIRL, FATHER and MOTHER – the predominant views are either of autism as a deficit or disorder (red) or as a disability or vulnerability (light red), reflecting the medical model. A smaller proportion emphasises treatments and interventions (green), while some samples link autism to violence or abuse (magenta).

**Figure 2.**
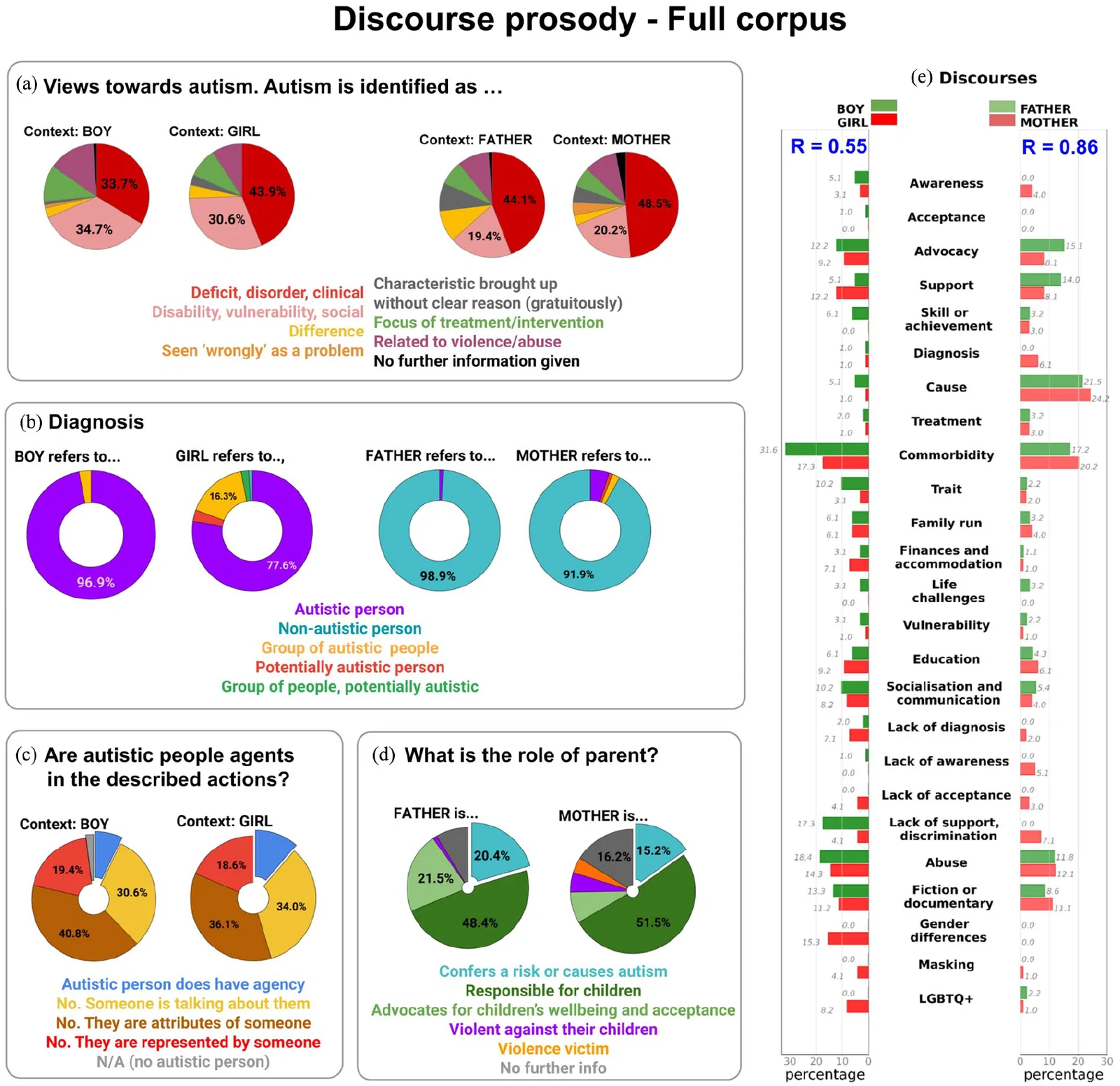
Annotation results for the full corpus. (a) General views towards autism (category I); (b) Diagnosis (category II); (c) Agency of the autistic person in described actions (category III); (d) Role of parents (category IV). (e) Histogram of discourse values for the BOY, GIRL, MOTHER and FATHER sets (category V).

The prevalence and rank order of these values for category I, which collectively underscore adversities associated with autism and autistic people, were generally comparable across all sample sets regardless of age and family role. In other words, newspapers similarly focused on adversities when referring to boys, girls, mothers, or fathers of autistic children.

More positive views of autism as a difference (yellow) were rare across all sets, with a slight, yet non-significant, prominence in the FATHER set (FATHER-MOTHER comparison, BIC = −1.73, no difference). In this set, text samples often highlighted unique cognitive and perceptual styles of autistic people, sometimes exaggerating these characteristics. Examples include, ‘“We find [. . .] why fathers and grandfathers of children with autism are over-represented in STEM fields”, he said . . .’ [*Telegraph*, November 2015] and ‘A[mbitious] US drama about a fallen-from-grace father [. . .] and his autistic son, who sees patterns in life that no one else can. It’s a magical show at times . . .’ [*Mail*, July 2016].

### Category II: diagnosis

Figure 2, panel B, illustrates the diagnostic status of collocates in the BOY, GIRL, FATHER and MOTHER text samples, revealing how newspapers associate autistic identity with different genders and family roles. In the BOY and GIRL samples, collocates (e.g. *boy, son, lad, girl, daughter*) predominantly referred to autistic people (purple). In contrast, in the MOTHER and FATHER samples, collocates (e.g. *mother, mum, dad*) primarily referred to neurotypical people (cyan). This suggests that newspapers often depict autistic children, while adults are typically mentioned in relation to an autistic child and are usually not autistic themselves.

Two gender-related differences emerged within family roles. First, GIRL samples more frequently referred to groups of autistic people (yellow) compared to BOY samples (BIC = 4.49, positive evidence). Many GIRL samples were from articles discussing autism in females, often portraying autistic girls as a newly discovered category with unique characteristics and challenges. For example, ‘. . . According to [. . .], one of the main issues that girls with autism face is the onset of puberty . . .’ [*Mail*, April 2012].

Second, MOTHER samples referred to autistic people or groups more often than FATHER samples (BIC = 2.56, positive evidence). This suggests newspapers occasionally mentioned autistic mothers but rarely referred to autistic fathers. Some references to autistic mothers aimed to inform readers or raise awareness, including them in the discussion of topics closely related to their intersectional identity. For instance, ‘Evidence matters to me because as an autistic woman and mother to autistic children, I am incredibly frustrated that the science telling us vaccines do not cause autism is being regularly ignored’ [*Times*, June 2017]. However, other references problematised the existence of autistic mothers, such as, ‘Society thinks that autistic mothers are, first and foremost, a safeguarding issue’ [*Guardian*, April 2017].

### Category III: agency

Category III, illustrated in panel C of Figure 2, was applied only to the BOY and GIRL sets to examine the extent to which autistic children were presented as agents in the actions described in the article (blue), were merely discussed by others (yellow), were described as attributes of their carer (brown), or were represented by someone else (red). The distribution indicated that both boys and girls were rarely depicted as having agency in the described actions in newspapers (BOY: 7.14%; GIRL: 11.22%; BIC = −4.38, no difference). The proportions of the other three categories were also comparable for both sets.

In the few instances where autistic boys and girls were agents, the articles often described extraordinary achievements or overcoming barriers related to autism. Examples include, ‘An autistic boy of four who hardly ever speaks burst into song on a supermarket trip’ [*Sun*, April 2020] and ‘An 11 year-old girl with autism has raised hundreds of pounds to help young people with the condition learn to swim’ [*Mirror*, April 2017]. Other actions related to stereotypes associated with autism, such as obsessions, vulnerability and needs. Examples include, ‘An autistic boy with an obsession for vacuums is heartbroken after thieves stole his prised Henry hoover’ [*Sun*, June 2018] and ‘Autistic girl, 11, has “cried every day” since “best friend” therapy dog stolen’ [*Star*, December 2019]. Finally, some actions involved social interactions, often depicted humorously, and highlighting attributes like honesty and awkwardness associated with autism. For instance,

When the Princess Royal turned up at one of the charities she supports to meet a gathering of disabled and autistic children, a small girl boldly informed her: ‘You don’t look much like a princess’. Anne didn’t miss a beat. ‘Good’, she replied. ‘That is very reassring’. [*Times*, July 2020]

### Category IV: role of parent

Panel D in Figure 2 illustrates the annotation results for category IV, which focuses on the role of mothers and fathers associated with autism, even if they are not autistic themselves. Most samples in this category depicted parents as caregivers for autistic children (dark green). For example, ‘Two mothers of severely autistic young adults also gave evidence on how the outbreak had harmed their children’s mental health’ [*Independent*, May 2020]. A notable proportion of samples also showed parents engaging in activism or advocacy for autistic people (light green). Examples include, ‘[. . .] is the mother of two autistic boys, aged 21 and 18, and has been married for 24 years. She is opening her second school for autistic children . . .’ [*Telegraph*, February 2011], and ‘Crowdfunding is being used to expand an app developed by a father to help his autistic son’ [*Telegraph*, April 2014]. These advocacy roles were slightly more common in the FATHER compared to the MOTHER set (BIC = 0.83, anecdotal evidence).

A notable percentage of quotes portrayed parents as conferring risk or causing autism through genetic predispositions or lifestyle choices (cyan). This blame was equally assigned to fathers (20.4%) and mothers (15.2%) (BIC = −4.78, no difference). However, the blame reasons differed: in the FATHER samples, most references linked autism to parental age, while the MOTHER samples mentioned a wider range of risk factors including prenatal lifestyle, labour conditions, substance abuse, nutrition and exposure to chemicals. An illustrative example is, ‘It also studied children in specific higher risk groups for the development of autism (sibling with autism, low birth weight, older fathers or mother smoking in pregnancy, etc)’ [*Telegraph*, March 2019].

Finally, mothers were more frequently mentioned in the context of violent events, either as victims or perpetrators (purple), compared to fathers (BIC = 1.61, anecdotal evidence). For instance, ‘The distraught mother of a severely autistic boy who has violent outbursts broke down yesterday, pleading: “I cannot live another day with my son”’ [*Mirror*, September 2017].

### Category V: discourses

Panel E in Figure 2 shows the percentages for 25 underlying discourses identified in text samples. These discourse values, presented using butterfly histograms (green for male, red for female), are not mutually exclusive, as a text sample could be assigned multiple values. The histograms display discourses related to children on the left and parents on the right. Frequent discourse values include comorbidity, cause (for FATHER and MOTHER), advocacy and abuse, corroborating the prevalence of medicalised views of autism, discussions of violence or abuse, and portrayals of parents as responsible for or advocating for their autistic children.

The comparison of discourse distributions across different sample sets (Supplementary Figure 1A) indicated that the similarity of discourses was less pronounced between BOY and GIRL (*R* = 0.55, *p* < 0.01) than between MOTHER and FATHER (*R* = 0.86, *p* < 0.001) (statistically significant difference, *z* = 2.24, *p* = 0.03). Differences between BOY and GIRL sets were driven by three discourses unique to the GIRL set: gender differences (BIC = 15.50, strong evidence), masking (BIC = 0.25, anecdotal evidence) and LGBTQ+ (BIC = 5.79, strong evidence).

Most GIRL-specific discourses fell into two types of news story. The first type discussed how autism in girls differs from boys and the challenges in diagnosing autism in girls due to camouflaging. For example, ‘Girls with autism are not being diagnosed because they are adept at camouflaging their behaviour in an effort to try to fit in, according to a new study . . .’ [*Guardian*, October 2020]. The second type of story focused on transitioning, particularly for children assigned female at birth, covering debates over whether transitioning should be allowed, referencing children with female pronouns and words. For example, ‘But her parents feel parental responsibility should take precedence until she is at least 16. They also believe their daughter may be on the autistic spectrum . . .’ [*Mail*, October 2016].

There was a higher proportion of discourses about lack of support or discrimination for BOY compared to GIRL (BIC = 3.38, positive evidence), often in stories highlighting how institutions fail autistic boys. For instance, ‘Mum says school “singled out” son. A boy with autism was made to wear a fluorescent jacket in the playground so he stood out from other children’ [*Mail*, March 2018].

In the FATHER and MOTHER sets, mothers were presented as facing more challenges related to supporting or accepting autistic children. Relevant discourses, such as lack of support and discrimination (BIC = 4.00, positive evidence) and lack of awareness (BIC = 1.35, anecdotal evidence) were unique to the MOTHER set. The same applied to discourses on diagnosis or the lack of diagnosis (BIC = 2.67, positive evidence), often highlighting the slow diagnostic process. For example, ‘One teenager left mainstream education after a decade-long battle for an autism diagnosis. Her mother, [. . .], said the teen decided she wanted to leave school aged 15’ [*Mail*, November 2018].

### Changes over time

Figure 3 presents the annotation results for the Early, Middle and Recent subcorpora. For category I (general views towards autism, shown in panel A) and category IV (agency in described actions, shown in panel D), the results focus on the GIRL set. Overall, the proportion and rank order of values in these categories was comparable to boys (for BOY results, see Figure 10 in Karaminis et al., 2023), and their distribution did not exhibit consistent changes over time.

**Figure 3.**
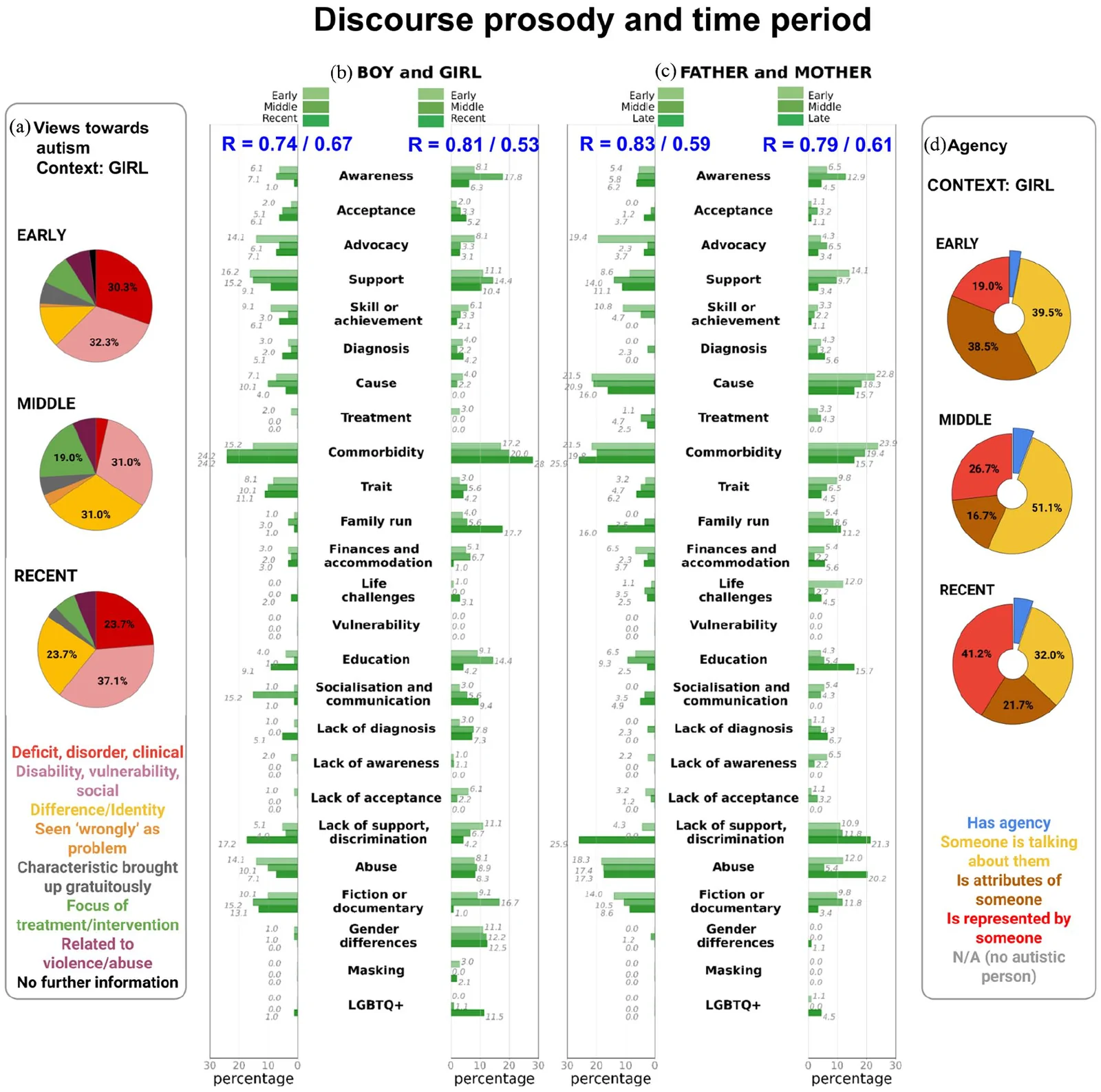
Annotation results for the Early, Middle and Recent subcorpora. (a) General views towards autism for the GIRL sample set (category I) in the BOY sample set; (b and c). Discourse values (category V) for the BOY, GIRL, MOTHER and FATHER sample sets; (d) Agency for the GIRL sample set (category IV).

In category V, panels *C* and *D*, correlation coefficients comparing discourses across sample sets tended to be higher for comparisons between the Middle and Recent subcorpora than for comparisons between the Early and Middle subcorpora across all four sets. This trend was more pronounced for the GIRL set (Early vs Middle, *R* = 0.81; Middle vs Recent, R = 0.53; non-significant trend, *z* = 1.77, *p* = 0.077) and the MOTHER set (Early vs Middle, *R* = 0.83; Middle vs Recent, R = 0.59; non-significant trend, *z* = 1.72, *p* = 0.085), suggesting that representations of autism in the Recent subcorpus for GIRL and MOTHER contexts were slightly differentiated from the earlier subcorpora.

For the GIRL set, the LGBTQ+ discourse first emerged in the Recent subcorpus (Middle vs Recent: BIC = 3.89, positive evidence), reflecting recent stories on gender dysphoria and transitioning in autistic children assigned female at birth. Several documents also discussed female autistic characters in TV series and movies, as well as a documentary on an autism boarding school for girls ( ‘Girls with Autism’; aired 2015), resulting in higher fiction/documentary values in the Middle than the Recent corpus (BIC = 10.39, very strong evidence). These texts often highlighted differences between autistic girls and boys and portrayed autism through stories of adversities and challenges. For example, a TV column alluded to a popular documentary, ‘. . . An insight into the UK’s only state-run boarding school for girls with autism reveals how the debilitating condition affects girls differently from boys’ [*Mail*, July 2015] and ‘This documentary shows how the condition manifests itself differently in every pupil–from Katie (above), 16, who is obsessed with boys, to Year 7 student Abigail, who refuses to speak . . .’ [*Sun*, July 2015].

Another subtle change over time in the representation of girls was the emergence of articles where girls are mentioned as part of a family with multiple autistic members (Middle vs Recent: BIC = 0.94, anecdotal evidence). For example, ‘. . . three-year-old daughter [. . .] has autism just like her older brother and sister’ [*Sun*, February 2020].

### Differences in reporting style

Figure 4 presents the annotation results for Broadsheets and Tabloids. In category I (general views towards autism), broadsheets more frequently adopted a difference-based view, suggesting more positive views towards autism, particularly in the GIRL set (BIC = 5.20, positive evidence; BOY: BIC = -1.31, no difference). In contrast, tabloids had more gratuitous references to autism, indicating a sensationalist style, particularly in the BOY set (BIC = 3.73, positive evidence; GIRL: BIC = 0.92, anecdotal evidence).

**Figure 4.**
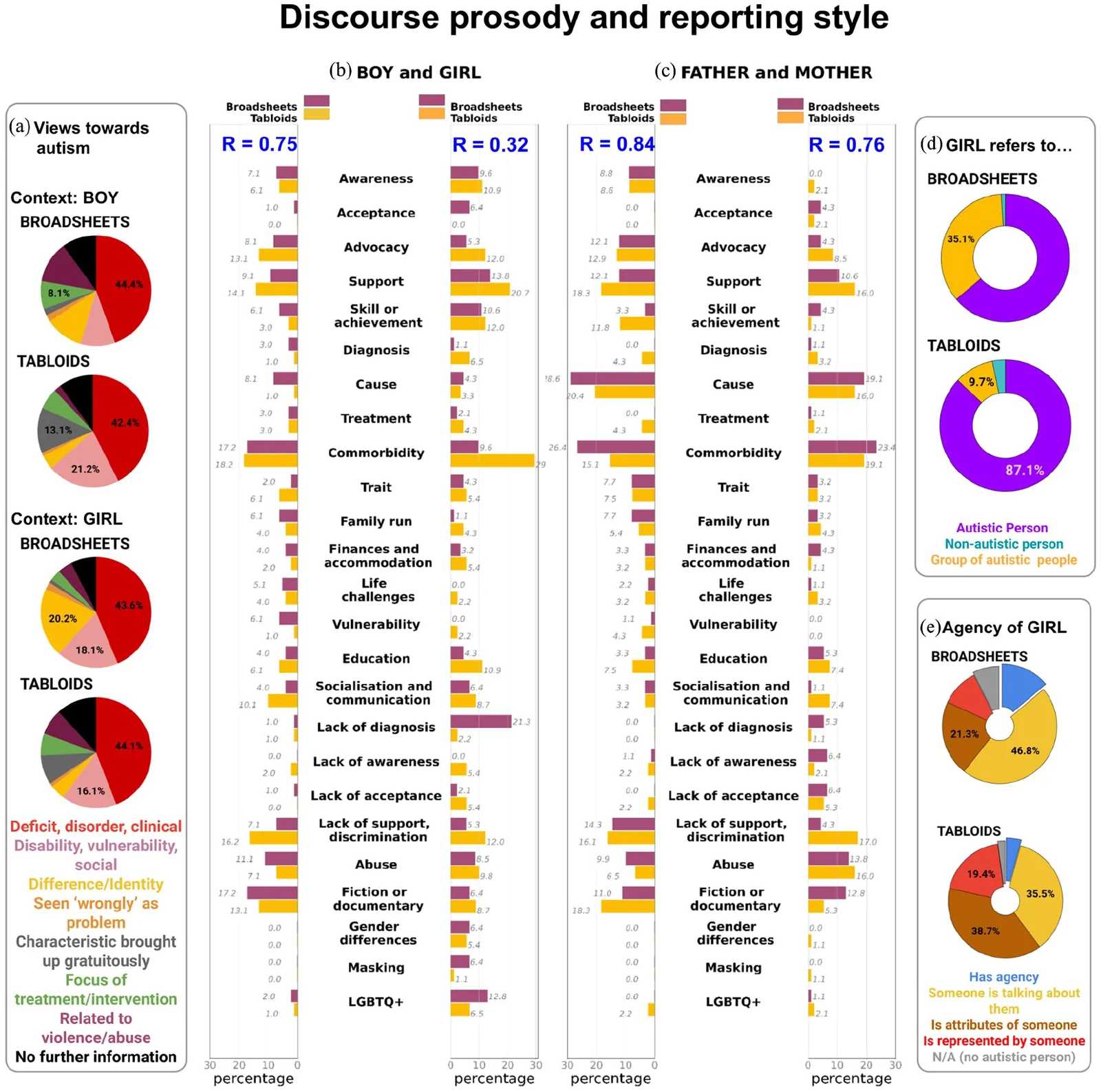
Annotation results for Broadsheets and Tabloids. (a) ‘General views towards autism’ (category I) for the BOY and GIRL sample sets; (b and c) Discourses values (category V) for the BOY, GIRL, MOTHER and FATHER samples; (d) Diagnosis (category II) for GIRL; (e) Agency (category III).

In category II (diagnostic status, subplot E), broadsheets more often referred to autistic girls in the plural compared to tabloids (BIC = 8.84, strong evidence), and discussed female autism within thematic frames addressing societal issues. For example, ‘These women are coming to prominence now because there’s more information on autism in girls and women on the internet, . . .’ [*Times*, December 2016]. In contrast, tabloids focused on actions and events related to an individual using episodic frames: ‘Autistic girl’s 10 hours in a cell–because police wrongly thought she was drunk . . .’ [*Mail*, January 2013].

In category III (agency, subplot F), autistic boys and girls had agency in a small percentage of text samples, with comparable percentages in both broadsheets and tabloids (BOY: BIC = −3.96, no difference; GIRL: BIC = −0.40, no difference). However, autistic boys were more often portrayed as being represented by someone else in tabloids compared to broadsheets (BIC = 3.69, positive evidence); this difference was not found for girls (BIC = −2.73, no difference).

For category V, when comparing discourses in broadsheets and tabloids, correlation coefficients suggested more pronounced reporting-style-related differences for girls than for boys (BOY: *R* = 0.73; GIRL: R = 0.30; marginally significant difference: *z* = 1.95, *p* = 0.05). By contrast, the discourses were comparable between broadsheets and tabloids for both MOTHER differences. A notable difference for the GIRL set was the higher proportion of lack of diagnosis discourses in broadsheets (BIC = 11.48, very strong evidence). For example, ‘The most common misconception about autism is that girls don’t have it’ [*Telegraph*, July 2015]. Conversely, tabloids had a higher proportion of co-morbidities discourse (BIC = 4.58, positive evidence), often combined with discourses on abuse and lack of support. For example, ‘Beth, a 17 year-old autistic girl self-harming and growing obese from inactivity after being locked up for 21 months and fed through a hatch in a padded cell’ [*Mail*, October 2018].

For the MOTHER and FATHER sets, differences in individual discourse values were generally small and not statistically significant, except for discourses on lack of support and discrimination, which were higher in tabloids for the MOTHER set (BIC = 2.47, positive evidence). For example, ‘A young mum whose autistic daughter set her kitchen on fire has pleaded: “I can’t cope, I need support”’ [*Mirror*, September 2017].

### Political orientation

Figure 5 shows annotation results for Left- and Right-leaning newspapers. Regarding category I (General views towards autism), the proportions of disability-based and difference-based views were similar across political orientations. However, uniquely in the FATHER set, left-leaning newspapers had more text samples focusing on treatments, interventions and support offered to children (BIC = 10.28, very strong evidence). For example, ‘The proud father of a boy with autism says a love for magic tricks has changed his son’s life’ [*Mirror*, January 2020].

**Figure 5.**
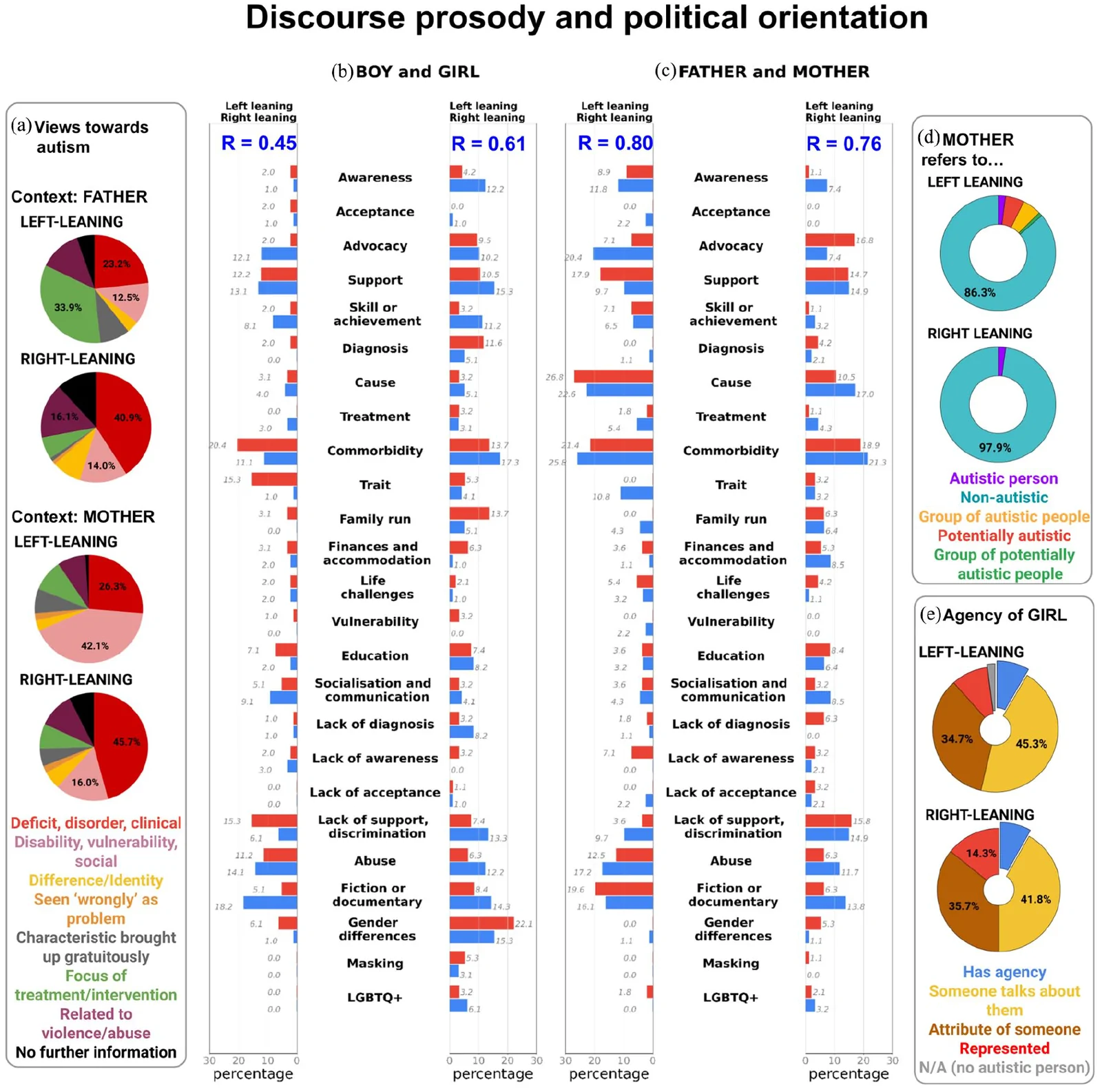
Annotation results for Left-leaning and Right-leaning newspapers. (a) General views towards autism (category I) in the FATHER and MOTHER sample sets; (b and c) Discourses (category IV) in the BOY, GIRL, MOTHER and FATHER samples; (d) Diagnosis (category II) for the MOTHER sample set; (e) Agency (category IV) in the GIRL sample set.

In categories II (diagnosis), III (agency) and IV (role of parent), the differences between left- and right-leaning papers were slight. Left-leaning newspapers discussed autistic mothers in the plural more often than right-leaning ones (BIC = 1.7, anecdotal evidence) and tended to present a higher proportion of autistic boys with agency in the described actions (BIC = 0.7, anecdotal). Conversely, right-leaning newspapers tended to depict mothers as responsible for someone in need of care more frequently than fathers (BIC = 1.41, anecdotal).

For category V (Discourses), political orientation differences tended to be more pronounced for the BOY (*R* = 0.45) and GIRL (R = 0.61) sets, with higher coefficients for the MOTHER (R = 0.80) and FATHER (R = 0.76) sets. However, differences between correlation coefficients for different genders were not statistically significant.

In the BOY set, left-leaning newspapers had a higher proportion of the autism traits discourse (BIC = 9.56, strong evidence), highlighting both negative and positive characteristics (e.g. aptitude in STEM topics). In addition, right-leaning newspapers had a higher proportion of advocacy discourses (BIC = 2.59, positive evidence), featuring stories on public figures advocating for their children and autistic people in general, as well as more references to fiction and documentaries (BIC = 2.39, positive evidence). These differences did not characterise the portrayal of autistic girls. For the GIRL set, there were numerical differences in discourse values between left- and right-leaning newspapers (e.g. more ‘awareness’ discourses in right-leaning, and more discourses on autism running in the family in left-leaning), but these were not statistically significant (BICs < 0).

## Discussion

In this study, we systematically analysed how British newspapers’ representations of autism differed by gender and family roles. We also examined how these variations evolved over time and across different publications. Our approach contrasted autism representations within four intersectional contexts of gender and family role.

On one hand, we found a notable uniformity across all four contexts in the stereotypical representation of autism, focusing on adversities and adopting a deficit-based rather than a difference-based perspective. Autistic boys and girls were often portrayed peripherally rather than centrally, from the perspectives of their mothers and fathers representing them or advocating for them. Mothers and fathers were highlighted for their efforts and challenges, but sometimes effectively blamed for their child’s autism. In other words, the key biases in the representation of autism in British newspapers identified by Karaminis et al. (2023) appear consistent in this follow-up study regardless of gender or family role. This indicates the robustness of these biases, which were also reported in other studies on the press portrayals of autism (Holton et al., 2014; Mittmann et al., 2024; Yu & Farrell, 2020), as well as studies on the representation of autism in fictional media (Jones et al., 2023; Mittmann et al., 2024; Nordahl-Hansen et al., 2018) and more recently social media (Corti et al., 2022; Gabarron et al., 2022; Skafle et al., 2021).

On the other hand, clear gender-related disparities emerged in the depiction of autistic children in press discourse in our study. Autistic girls were often referred to as a newly identified group, explicitly contrasted against the traditional autistic boy baseline, and frequently associated with specific themes such as difficulties in diagnosis and camouflaging. Girls appear marginalised in the autism discourse, depicted as either a recent discovery or previously overlooked. Their collective framing, unlike the more individualised portrayal of autistic boys, may obscure their unique experiences (Pearson & Rose, 2021).

These findings on the way in which autistic girls are framed in the British press, reveal yet another mechanism for male-gender bias in newspapers. This mechanism complements earlier observations that autistic girls are mentioned much less frequently than autistic boys in British newspapers (Karaminis et al., 2023). As a result of these two mechanisms, namely newsworthiness and framing, girls face ‘double invisibility’, with their specific experiences neither widely discussed nor well-represented in newspapers. It is likely that a similar ‘double invisibility’ exists for autistic girls across other media; however, existing studies tend to focus more on quantitative differences (Dean & Nordahl-Hansen, 2022) than on qualitative aspects.

The disparities in the representation of autistic boys and girls reflect entrenched gender stereotypes and societal biases towards gender and disability (Barnett, 1997; Davis & Hines, 2020; Han et al., 2022; Witt, 1997). For example, stereotypes suggest boys should be active and noisy, while girls should be quieter and more communal (Koenig, 2018; Martin, 1995). Depictions of autistic boys often emphasise external behaviours and challenges, such as disruptive behaviours or educational difficulties and, more rarely, their specific gifts and capabilities. In contrast, autistic girls are portrayed with internalising behaviours and social camouflage challenges, which may not be obvious to educators or caregivers (Geelhand et al., 2019), making diagnosis and intervention more difficult.

Furthermore, the disparities in the representation of autistic boys and girls partly reflect historical gender biases in autism research, influenced by theories like the ‘extreme male brain’ and the ‘empathising-systemising’ theory, as well as the female autism phenotype. Recently, autism research has aimed to remove participation barriers and improve inclusion for underrepresented groups (Duhon et al., 2023; Nicolaidis et al., 2019). Studies mapping research priorities for the autism community emphasise understanding intersecting autistic identities and supporting their diverse needs (Cage et al., 2024; Pellicano et al., 2014; Putnam et al., 2023). Despite progress and efforts to disseminate research through press coverage (Longo & Hand, 2023), there is likely still a lag between advancements in research and their reflection in newspapers. This delay suggests that gender disparities in the understanding of autism representation may persist in newspaper discourse for some time.

Going beyond binary gender categories and considering transgender autistic children, particularly those assigned female gender at birth, we found that press coverage often emphasised parental concerns regarding the role of autism in their gender identity. This framing reflects a poor translation of actual research findings by reducing the issue of the overlap between autism and gender non-conformity to a question of whether transgender children ‘just have autism’ (van der Miesen et al., 2018). Furthermore, the language used often failed to affirm the child’s chosen gender, risking perceived misgendering. These portrayals do not promote understanding of the unique experiences of transgender autistic youth (Strang et al., 2018) and may reinforce transphobic stereotypes.

Our findings demonstrate that gender-related biases in press representations of autism extend to the immediate families of autistic children. The child’s autism was attributed to the mother with a more diverse set of possible pathways than those suggested for fathers, including genetic, behavioural and lifestyle factors, while mothers were more often associated with violent incidents involving their children. Mothers of autistic children were more often mentioned in newspaper articles than fathers, with this motherhood often discussed negatively or as a liability.

These portrayals rely on stereotypes of mothers as primary caregivers (Hodges & Park, 2013) and bearers of responsibility for their child’s physical and mental health (Lutz, 2024), while reflecting a limited understanding of fathers’ experiences (Seymour et al., 2017). Newspapers stigmatise mothers by suggesting a propensity towards crisis or failure and subject them to heightened ethical scrutiny. This is particularly pronounced when stereotypes about mothers are coupled with negative stereotypes about autistic people (Adams et al., 2021; Pohl et al., 2020).

The findings on changes over time indicate an evolving narrative around autism that intersects with gender and family roles. Notably, texts referring to girls and mothers showed a shift in discourses from early to recent subcorpora, with emerging topics such as LGBTQ+ issues and autism representation in documentaries. These shifts reflect a broadening of autism-related topics and increased visibility of autistic females. However, they also highlight the stereotypical nature of these representations, which can reinforce invisibility and negative outcomes (Pearson & Rose, 2021).

In terms of reporting style, the differences between broadsheets and tabloids were particularly pronounced in the portrayal of autistic girls, showcasing contrasting narratives. Broadsheets framed autism in girls within a thematic and societal context, potentially offering a more nuanced understanding based on research findings. However, these narratives still reinforced stereotypes that portray autism in girls as secondary or less prominent compared to autism in boys. Conversely, tabloids often featured sensational stories about individual autistic girls, emphasising adversities and a deficit-based perspective to captivate readers and boost circulation (Molek-Kozakowska, 2013). In terms of political orientation, our findings suggested that differences between left and right-leaning newspapers in framing autism were more subtle, and were often focused on the portrayal of boys. For example, coverage of autistic boys in left-leaning newspapers emphasised autism characteristics, both challenges and strengths, whereas right-leaning newspapers tended to feature stories about public figures advocating for autism, as well as references to autism-related documentaries and fiction.

Collectively, our findings demonstrate a bias in the ways that newspapers portray female autism as less significant, and place mothers of autistic children under more critical scrutiny than fathers. Coupled with the higher frequency of discussions about boys and mothers than girls and fathers (Karaminis et al., 2023), this bias reinforces gender inequalities in the public perceptions of autism and entrenches stereotypes about gender and family roles within autism narratives.

This study is the first, to our knowledge, to examine and contrast press representations of autism intersectionally; however, it has its limitations. First, many intersections, such as age (see O’Connor & Downey, 2024), race and cultural background, need further exploration (Botha & Gillespie-Lynch, 2022; Mallipeddi & VanDaalen, 2022). For studies of these intersections using corpus-based approaches to be viable, the granularity and richness of data must be balanced with inclusivity. In addition, in characterising changes over time in the representation of intersectional groups of autistic people, it would be beneficial to consider the impact of critical events rather than relying solely on arbitrarily defined seasonal subcorpora. Future studies could, therefore, address the effects of events such as COVID-19 (see Corti et al., 2022) on the intersectional autism representations or the impact of Greta Thunberg’s activism on the portrayal of autistic girls (Skafle et al., 2021).

Second, like many studies on newspaper representations, the influence of these portrayals on public beliefs about autism warrants further exploration. Further studies should consider their relative impact and interplay with demographic factors, personal views, critical thinking ability and the influence of other news sources. Our ongoing work addresses this issue empirically.

Third, although community members were involved in the analysis, it is important to corroborate these results with stronger models of research participation (Fletcher-Watson et al., 2019; Nicolaidis et al., 2019; Pellicano et al., 2017) including female and gender-diverse autistic people (see also Karaminis et al., 2024). In addition, future research should explore the impact of media portrayals on the self-identity and self-esteem of autistic people (Cage et al., 2018; Han et al., 2022), considering how biases in media narratives interact with internalised stigma (Bachmann et al., 2019) within underrepresented groups and affiliate stigma in parents (Kinnear et al., 2016).

Autistic people and families should be partners in future research aimed at developing effective interventions to educate media professionals about the diversity of the autistic experience, for which the findings of this study may be particularly relevant. Input from diverse groups within the autism community could help shape more nuanced and inclusive media representations. Such initiatives could ultimately influence positively the public discourse, promoting visibility, acceptance and support for all members of the autism community.

## Supplemental Material

sj-docx-1-aut-10.1177_13623613241303547 – Supplemental material for Gender and family-role portrayals of autism in British newspapers: An intersectional corpus-based study Supplemental material, sj-docx-1-aut-10.1177_13623613241303547 for Gender and family-role portrayals of autism in British newspapers: An intersectional corpus-based study by Themis Karaminis, Costas Gabrielatos, Ursula Maden-Weinberger and Geoffrey Beattie in Autism
