# Supplementary material for: Gender and family-role portrayals of autism in British newspapers: An intersectional corpus-based study
Source: Autism. 2024 Dec 11;29(5):1285–302. doi: 10.1177/13623613241303547 (PMC13687621; doi:10.1177/13623613241303547)
Supplement: Supplementary file 1 [file sj-docx-1-aut-10.1177_13623613241303547.docx]

# Supplementary Figures

## Supplementary Figure 1

Representational similarity heatmaps, summarising the similarity of discourses based on the 25 values of category V. Higher values are shown with bolder red shades and indicate higher similarity, with 1.00 corresponding to perfect agreement. A. Pairwise comparisons of discourses for different sample sets in the full corpus and the seven subcorpora. B. Pairwise comparisons of discourses for a given gender and family role intersection in subcorpora with different reporting style, political orientation, and publication time.
